# Supplementary material for: Effects of exercise modalities on heart rate recovery and its association with cardiometabolic risk in adolescents with overweight or obesity
Source: PLoS One. 2025 Nov 7;20(11):e0336371. doi: 10.1371/journal.pone.0336371 (PMC12594340; doi:10.1371/journal.pone.0336371)
Supplement: Attachment — Submitted filename: General comments.docx [file pone.0336371.s002.docx]

Comments

The manuscript entitled *“Effects of different types of exercise training on heart rate recovery in adolescents with overweight and obesity”* addresses a highly pertinent and clinically important research question by investigating not only the differential impact of aerobic (AE), resistance (RE), and combined training on heart rate recovery (HRR), but also by exploring the associations between HRR and key indicators of body composition and cardiometabolic risk. This dual focus adds depth and translational value to the study, especially considering the rising frequency of obesity-related health issues in adolescents and the crucial role of autonomic function as an early indicator of cardiovascular risk, as measured by HRR. Furthermore, the inclusion of body composition and metabolic variables broadens the study’s clinical implications and supports a more integrated understanding of the physiological adaptations to exercise in overweight and obese adolescents.

However, to fully realize the potential of this contribution, several important issues merit further clarification. Most notably, the standardization and monitoring of exercise duration and intensity across training groups need to be addressed more thoroughly to ensure comparability of intervention effects. Additionally, a more detailed discussion of the physiological mechanisms that may underpin the differential HRR responses to AE, RE, and combined training would enhance the manuscript’s interpretive depth and theoretical contribution. The following will elaborate on these aspects in detail, hoping to provide a useful reference for the further optimization of the manuscript.

1. The current title provides a fundamental overview of the study's emphasis but does not fully reflect the dual aims mentioned in the manuscript, namely the assessment of the association between heart rate recovery (HRR) and both body composition and cardiometabolic risk factors. To clarify the study's scope, I recommend changing the title to "Effects of Exercise Modalities on Heart Rate Recovery and Its Association with Cardiometabolic Risk in Overweight and Obese Adolescents."

The revised title clearly states the intervention's comparative character as well as the correlational analysis including HRR and cardiometabolic outcomes, boosting both specificity and relevance for potential readers.

2. To strengthen the clarity and interpretability of your findings, I recommend that you include percentage change for pre- to post-test analyses, in addition to reporting exact p-values and effect sizes (e.g., partial eta squared, η²) for all outcomes where significant differences were found. Since your analysis is based on repeated measures ANCOVA, these additional statistical details are crucial for conveying not only statistical significance but also the magnitude and practical relevance of the observed effects. Consistent reporting of these values in both the abstract and the main results section will enhance the scientific rigor, transparency, and overall impact of your manuscript.
3. In the methodology section, it is noted that the control group was observed for three months, while participants in the AE and RE groups underwent intervention for either three or six months, and the combined training (CT) group participated for the full six months. This variation in intervention duration across groups raises concerns regarding the comparability of exercise modalities. Even if time is statistically treated as a confounding variable, the unequal exposure duration introduces a potential bias that may affect the interpretation of outcomes. To ensure valid comparisons between exercise types, intervention duration should ideally be standardized across groups or more clearly justified and accounted for in the analysis and discussion.
4. To further demonstrate the rigorous scientific methodology and replicability of your study, it is recommended that you provide the brand name, manufacturer, and country of origin for all essential equipment utilized, such as those for height, weight, lipid profile measurement, and treadmill-based testing or training. Please add information about the validity and reliability of these instruments from past validation studies. This information is critical for assessing measurement accuracy and consistency, as well as allowing other researchers to reproduce your processes or compare results across studies. Incorporating these elements within the Methods section will increase the scientific credibility of your work.
5. As the primary objective of your study is to examine the effects of different types of exercise training on heart rate recovery, it is important to clearly present both within-group (pre–post) and between-group effects in the Results section. While the manuscript discusses pre–post analyses result and between group effect in the text, there is currently no table provided that summarizes these findings. To improve the clarity, transparency, and interpretability of your results, I strongly recommend including two tables: one showing pre–post changes within each group, and another summarizing the between-group effects, with appropriate statistical metrics such as percent change, mean differences, confidence intervals, p-values, and effect sizes (e.g., η²). This will significantly enhance the readability and scientific rigor of your manuscript.
6. In the Discussion section, the physiological rationale for the observed improvement in heart rate recovery (HRR) warrants further elaboration. While parasympathetic reactivation and sympathetic withdrawal are briefly mentioned, the authors should more thoroughly explain these mechanisms and explicitly relate them to the specific exercise interventions used. For instance, detailing how aerobic exercise uniquely promotes autonomic adaptations would provide a clearer interpretation of the findings. Integrating this explanation would enhance the discussion by linking the results to underlying physiological processes and aligning them with existing research. And also the reported association between improvements in HRR (at 2–5 minutes) and VO₂peak is an important finding, yet its practical significance is not fully elaborated. How might these relationships inform future training prescriptions or screening strategies for cardiovascular risk in adolescents? Including such implications would enhance the translational value of the findings.
