# Supplementary material for: Effects of exercise modalities on heart rate recovery and its association with cardiometabolic risk in adolescents with overweight or obesity
Source: PLoS One. 2025 Nov 7;20(11):e0336371. doi: 10.1371/journal.pone.0336371 (PMC12594340; doi:10.1371/journal.pone.0336371)
Supplement: Attachment — Submitted filename: last Comment.docx [file pone.0336371.s004.docx]

**Comment:**
Thank you for the revision. While I acknowledge the additional details regarding the anthropometric equipment and appreciate the clarification about the lipid analyses being conducted in a certified laboratory, I still have some reservations about the incomplete information concerning the instruments used for cardiometabolic measurements. Providing at least general specifications or citing validation references for these instruments would further strengthen the methodological transparency.
Nevertheless, I am satisfied with the other revisions and overall improvements made in response to this comment.
