# Supplementary material for: Effects of exercise modalities on heart rate recovery and its association with cardiometabolic risk in adolescents with overweight or obesity
Source: PLoS One. 2025 Nov 7;20(11):e0336371. doi: 10.1371/journal.pone.0336371 (PMC12594340; doi:10.1371/journal.pone.0336371)
Supplement: Attachment — Submitted filename: Reviewers comment - Response 2.docx [file pone.0336371.s006.docx]

**Reviewer #1:**

Thank you for the clarification. However, I still have some reservations regarding the instruments used, particularly for the cardiometabolic data. While certification of the laboratory by the CDC–NHLBI Lipid Standardization Program ensures quality control, it would still be helpful to provide at least general information about the analytical instruments or assay methods used (e.g., type of analyzer, assay principle, or manufacturer). This additional detail would further enhance methodological transparency and allow for better reproducibility and comparison across studies.

**Response:** We have elaborated on the methods used to assess the lipids in the manuscript methods as follows:

“A fasting blood sample was obtained for lipid analysis following a minimum 10-hour overnight fast. Fasting plasma lipids were measured using an Olympus AU400 Chemistry Analyzer in the Nutrition Laboratory of the University of Pittsburgh Graduate School of Public Health, which was certified by the Centers for Disease Control and Prevention-National Heart, Lung, and Blood Institute Lipid Standardization Program [26]. HDL was assessed using the two-reagent method. Triglycerides were determined enzymatically. Reagents used were obtained from Vital Diagnostics (Lincoln, RI). LDL and VLDL were calculated using the Friedewald equation [27]. For total and HDL cholesterol and triglycerides intra-assay coefficients of variation (CV) were 1.0%, 1.8% and 1.8% and inter-assay CV were 1.6%, 2.6%, and 3.7%, respectively.”

**Reviewer #2:**

Many thanks for the invitation to review this manuscript. In this paper the authors evaluate the effect of exercise modalities on heart rate recovery and its association with cardiometabolic risk in adolescents with overweight or obesity. The paper is well written and presents good elements of originality. I have no further comments.

**Response:** Thank you. We appreciate your contributions in this process to improve our manuscript.
